# Gafchromic film dosimetry of a new HDR ${\;}^{192}\text{Ir}$ brachytherapy source

**DOI:** 10.1120/jacmp.v17i2.6005

**Published:** 2016-03-08

**Authors:** Navid Ayoobian, Akbar Sarabi Asl, Hosein Poorbaygi, Mohammad Reza Javanshir

**Affiliations:** ^1^ Department of Nuclear Engineering Faculty of New Sciences and Technologies University of Isfahan Isfahan Iran; ^2^ Radiation Application Research School Nuclear Science and Technology Research Institute Tehran Iran

**Keywords:** IRAsource‐HDR ${\;}^{192}\text{Ir}$, brachytherapy, radiochromic film, dosimetry, 2D dose distribution

## Abstract

High‐dose‐rate (HDR) brachytherapy is a popular modality for treating cancers of the prostate, cervix, endometrium, breast, skin, bronchus, esophagus, and head and neck as well as soft‐tissue sarcomas. Because of different source designs and licensing issues, there is a need for specific dosimetry dataset for each HDR source model. The main objective of the present work is to measure 2D relative dose distribution around a new prototype ${\;}^{192}\text{Ir}$ source, referred to as IRAsource‐HDR, in PMMA phantom in the framework of AAPM TG‐43 and TG‐55 recommendations for radial distances of 0.5 cm to 4 cm. Radiochromic films (RCFs) Gafchromic EBT and HD‐810 were used for measurements. The dose rate constant, Λ, of the source was determined to be 1.084±4.6%,1.129±4.4%, and $1.112\pm 0.8\%\;{\text{cGyh}}^{-1}U^{-1}$ using EBTRCF, HD‐810 RCF, and Monte Carlo (MC) simulation, respectively. The results obtained in this study are in good agreement with previously published data for HDR interstitial ${\;}^{192}\text{Ir}$‐HDR sources with a maximum discrepancy of ±4.5%. An acceptable agreement (within ±2%) between MC calculations and RCFs measurements showed that HD‐810 RCF dosimetry is as good as EBTRCF, within HDR brachytherapy, and justifies the use of specific data for this new source. These data could be used as a benchmark for dose calculations in the conventional brachytherapy treatment planning systems.

PACS number(s): 87.56.bg

## I. INTRODUCTION

Dose distributions around HDR ${\;}^{192}\text{Ir}$ brachytherapy sources suffer from high dose gradients. Radiochromic film is a powerful 2D dosimeter, which exhibits useful characteristics including high spatial resolution, near tissue/water equivalence $\left(Z_{\text{eff},\text{EBT}}=6.98,Z_{\text{eff},\text{water}}=7.3\right)$,^1^, ^2^ the possibility of using in water, and lower sensitivity to visible light. In addition, it has been shown to have an energy independence response for HDR brachytherapy applications.^3^, ^4^

In recent years, many attempts have been made to perform the dosimetry of ${\;}^{192}\text{Ir}$‐HDR brachytherapy sources using radiochromic films (RCFs).^5^, ^6^, ^7^, ^8^, ^9^ Piermattei et al.^(5)^ performed experimental dosimetry of the MicroSelectron HDR ${\;}^{192}\text{Ir}$ source model 080950 (Elekta AB, Stockholm, Sweden), used for the brachytherapy of peripheral vessels in a water phantom with high sensitivity (HS) RCF. They reported the dose rate constant, Λ, of the ${\;}^{192}\text{Ir}$‐HDR source to be $1.11\pm 0.02\%\;{\text{cGyh}}^{-1}U^{-1}$. Sellakumar et al.^(7)^ measured dosimetric functions such as dose rate constant, radial dose functions, and 2D anisotropy function for HDR ${\;}^{192}\text{Ir}$ source V2, from the Nucletron MicroSelectron HDR unit using Gafchromic EBT films (Ashland Inc., Covington, KY) in water‐equivalent RW3 Solid Water phantom (PTW‐Freiburg GmbH, Freiburg, Germany).

They measured doses at radial distances of 0.5 cm to 5.0 cm with an interval of 0.5 cm and at polar angle between 0° to 180° in 10° intervals. This group obtained the dose rate constant, Λ, of the ${\;}^{192}\text{Ir}$‐HDR source equal to $1.1328\;{\text{cGyh}}^{-1}U^{-1}$. Their study indicates that the new Gafchromic EBT film is very sensitive and can be used to measure brachytherapy dosimetric functions with high resolution described in the American Association of Physicists in Medicine (AAPM) TG‐43.

The Nuclear Science and Technology Research Institute (NSTRI) in Iran is developing a new HDR ${\;}^{192}\text{Ir}$ brachytherapy source, model IRAsource‐HDR, as a substitute for available classical sources which has been under preclinical tests to be used in the remote afterloading system. Based on the AAPM TG‐43 and TG43‐U1 reports as well as the High Energy Brachytherapy Source Dosimetry (HEBD) Working Group requirements and licensing issues, the dosimetric characteristics of each new radioactive source type using experimental methods and Monte Carlo (MC) simulation should be determined.^10^, ^11^, ^12^ Thus, the purpose of this work is to determine the dosimetry parameters for an IRAsource‐HDR ${\;}^{192}\text{Ir}$ using both experimental (with Gafchromic EBT and HD‐810 RCFs) methods and MC simulation.

## II. MATERIALS AND METHODS

### A. IRAsource‐HDR ${\;}^{192}\text{Ir}$ brachytherapy source

**Figure 1:**
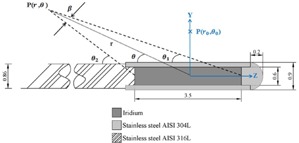
A schematic diagram illustrating the geometry of IRAsource. All dimensions are in millimeters.

Figure 1 illustrates schematic diagram of the IRAsource‐HDR ${\;}^{192}\text{Ir}$. This source model consists of a pure uniform iridium cylinder (density $22.42\;{\text{gcm}}^{-3}$) with an active length of 3.5 mm and a diameter of 0.6 mm. The active core is encapsulated in a stainless steel 304L tube (density $8.0\;{\text{gcm}}^{-3}$) leading to physical source dimensions of 0.9 mm in diameter and 4.63 mm of total length. The encapsulated end is hemispherical in shape with tip‐to‐pellet distance of 0.53 mm. A woven stainless 316L cable of 0.86 mm outer diameter is welded to the back end of the 304L tube. The active iridium core was produced from the neutron activation in the 5 MW TRR research reactor at the Atomic Energy Organization of Iran (AEOI).

Difference between IRAsource model and other similar ${\;}^{192}\text{Ir}$‐HDR sources in common use^13^, ^14^, ^15^, ^16^, ^17^ is listed in Table 1. All HDR sources have similar active length and active core diameter and are all encapsulated in stainless steel tubes.

**Table 1:** Physical dimensions (mm) of HDR ${\;}^{192}\text{Ir}$ source models and the IRAsource

*Source*	*Active Length*	*Total Length*	*Active Core Diameter*	*External Diameter*	*Capsule Thickness*	*Distal Capsule Thickness*
mHDR‐v2‐revise^(13)^	3.5	4.95	0.60	0.90	0.100	0.20
Flexisource Ir‐192^(14)^	3.5	4.60	0.60	0.85	0.090	0.65
GammaMed HDR 12i^(15)^	3.5	4.96	0.60	1.10	0.200	0.96
BEBIG‐HDR‐GI192M11^(16)^	3.5	4.90	0.60	1.00	0.200	0.84
Generic source^(17)^	3.5	5.00	0.60	1.00	0.200	0.60
IRAsource	3.5	4.30	0.60	0.90	0.150	0.25

### B. Gafchromic EBT and HD‐810 films

The Gafchromic EBT and HD‐810 (lot #: R2507H810, ISP Technologies Inc., Wayne, NJ, ED: 2017) RCFs were used in this work. The HD‐810 film (also known as DM‐1260) has a simple construction with only three layers; a waterproof polyester support layer (97μm), an active layer (6.5μm) and a surface layer (0.75μm) leading to a total film thickness of about 105 μm. Each pack of HD‐810 film contains five clear sheets with dimensions $8\times 10\;{\text{in}}^{2}$. The EBT film is composed of five layers; the outer layers are made of clear polyester (97μm) and the inner layers are composed of two active layers (17μm) surrounding a surface layer of approximately 6 μm thickness. In this study, the sheets of EBT and HD‐810 RCFs were cut into square pieces with dimensions of 3×3 and $5\times 5\;{\text{cm}}^{2}$, respectively. All measurement films used were from the same pack.

### C. Phantom assembly

**Figure 2:**
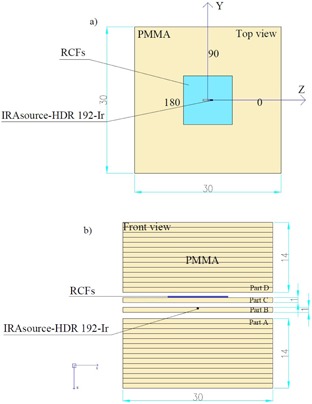
Schematic diagrams of the arrangement used to expose RCFs. The source groove is 2.0 cm in length and 1 mm in depth with a machining uncertainty of 0.005 cm. (a) Top view: the HD‐810 RCF is shown by the light blue rectangle positioned above the source. (b) Lateral view (the parts A, B, C, and D of PMMA slabs). All dimensions are in centimeters.

The water equivalent $\left(Z_{\text{eff},\text{PMMA}}=6.5\right)$ phantom with a physical density of $1.18\;{\text{gcm}}^{-3}$ and cubic dimensions of $30\times 30\times 30\;{\text{cm}}^{3}$ was used. This phantom consisted of PMMA slabs, each in $30\times 30\times 1\;{\text{cm}}^{3}$ dimensions. Arrangements of the source and RCFs in the phantom are shown in Fig. 2. Slab (part B) has a horizontal cubic groove with dimensions of $0.1\times 0.1\times 2\;{\text{cm}}^{3}$ created using a CNC machine and the IRAsource‐HDR ${\;}^{192}\text{Ir}$ was inserted into the groove. This slab was placed on a $30\times 30\times 14\;{\text{cm}}^{3}$ stack of PMMA slabs (part A). Each piece of HD‐810 and EBT RCFs was positioned horizontally above the HDR source as shown in Fig. 2. The source/film phantom assembly $\left(30\times 30\times 15\;{\text{cm}}^{3}\right)$ was then covered with additional $30\times 30\times 15\;{\text{cm}}^{3}$ PMMA stacks (parts C and D) to provide full backscatter conditions.^18^, ^19^

### D. Film scans

The EBT and HD‐810 RCFs were used to acquire responses of lower and higher doses from the HDR source, respectively. In order to deliver doses in the range covered by the film calibration curves (EBT: 0.5‐8 Gy, HD‐810: 10‐500 Gy) and with regard to experimental film size, irradiation times of 2.1 h and 182.4 h were used for EBT film at distances from 0.5 to 1.5 cm and HD‐810 at distances from 0.5 to 4 cm, respectively. EBT film strip $\left(3\times 3\;{\text{cm}}^{2}\right)$ was irradiated for shorter time to determine the near field, while larger HD‐810 $\left(5\times 5\;{\text{cm}}^{2}\right)$ film was irradiated for longer time in order to determine the far field. After the exposure, the films were stored for two days at room temperature in a lightproof envelope before processing and analysis, as recommended by the AAPM TG‐55 report.^20^, ^21^ The EBT and HD‐810 RCFs were then scanned using Microtek ScanMaker 9800XL (Microtek International Inc., Hsinchu, Taiwan) flatbed and HP ScanJet 6100C color scanner (HP Inc., Palo Alto, CA), respectively. The scanning resolution was set at 300 dpi in transmission mode and the resulting image file was saved in tagged image file format (.tiff). Prior to measurements, the light source in the scanner was turned on for 30 min and then several scans were made with empty scans to warm up sufficiently.

The lateral response artifact (LRA) of a scanner is of great importance in RCFs scanning (i.e., increases the dose up to 9% at ±5 cm lateral distance from the scanner axis for single channel dosimetry and 14 Gy exposure).^(22)^ By placing the RCFs at the center of the scanning bed in the same orientation and using triple‐channel method with transmission scan, this effect was mitigated significantly. After scanning RCFs, the moiré pattern or Newton's Ring‐like pattern may be observed. This effect was eliminated by using a plastic frame to keep film out of direct contact with the glass surface of the scanner.^(23)^ Also, the Callier effect influences the sensitivity of film response to the flatness of the film on the scan window.^(24)^ To mitigate this effect and to ensure all samples were flat, a pressed glass sheet was placed above the films. Thus, RCFs were digitized using 48‐bit mode, 16‐bit per color channel, and the average pixels were extracted to analyze with an image processing package, Fiji.^25^, ^26^

### E. Calibration of RCFs

To calibrate RCFs, 12 pieces of EBT in $1\times 1.5\;{\text{cm}}^{2}$ dimensions and 17 pieces of HD‐810 in $1.5\times 1.5\;{\text{cm}}^{2}$ dimensions were exposed to doses ranging from 0.5 to 9 Gy and 10 to 500 Gy, respectively. The irradiations were performed by a medical electron linear accelerator (Elekta 6 MV Linac, Novin Medical Radiation Institute, Tehran, Iran) for EBT RCF and Co‐60 therapy unit (type Picker V9, Secondary Standard Dosimetry Laboratory, Karaj, Iran) for HD‐810 RCF. Also, two pieces of EBT and HD‐810 RCFs were kept in the brachytherapy lab environment to measure the background dose.

### F. Data analysis

The optical density (OD) was defined as ${\text{log}}_{10}(S_{0}/S)$ where $S_{0}$ and *S* are the average pixels resulting from the Fiji software for unirradiated and irradiated films, respectively. Since the dose over the exposed area is not completely uniform, especially at the clipped edges of the films, only the average OD over a region of interest (ROIs, with the area of π cm^2^) at the center of each calibration film was obtained. The net OD values were obtained by subtracting the ODs of background from calibration or experimental films.

### G. TG‐43 dosimetry formalism

According to the TG‐43 formalism, the dose distribution around the cylindrical source, at a point P(r,θ) in Fig. 1, is calculated by:^10^, ^11^

(1) $$\dot{D}(r,\theta )=S_{k}\Lambda \frac{G(r,\theta )}{G(r_{0},\theta_{0})}g(r)F(r,\theta )$$

where $S_{k}$ is air‐kerma strength of ${\;}^{192}\text{Ir}$‐HDR brachytherapy source with units of $1U=\text{cGy}\;{\text{cm}}^{2}h^{-1}$, Λ is the dose rate constant expressed in cGy h^‐1^ U^1^, *G(r, θ)* is the geometry function, *G*($r_{0}$, *θ_0_)* is the geometry function at the reference position $\left(r_{0}=1\;\text{cm},\theta 0=90^{\circ }C\right)$, g(r) is the radial dose function, and F(r,θ) is the 2D anisotropy function.

The geometry function for a linear source with length L and subtended angle $\beta ,G_{L}(r,\theta )$, is calculated by:

(2) $$\beta =\frac{{\text{tan}}^{-1}(1/2r\text{sin}\theta +\text{cot}(\theta ))+{\text{tan}}^{-1}(1/2r\text{sin}\theta -\text{cot}(\theta ))}{L.r.\text{sin}(\theta )}$$

(3) $$G_{L}(r,\theta )=\{\begin{array}{ll} \frac{\beta }{L.r.\text{sin}(\theta )} & if\theta \neq 0^{0}\\ {\left(r^{2}-\frac{L^{2}}{2}\right)}^{-1} & if\theta =0^{0} \end{array}$$

The radial dose function, g(r), and the 2D anisotropy function, F(r,θ), for a linear source are expressed as follows:

(4) $$g(r)=\frac{\dot{D}(r,\theta_{0})}{\dot{D}(r_{0},\theta_{0})}\frac{G(r_{0},\theta_{0})}{G(r,\theta_{0})}$$

### H. Monte Carlo simulation

Experimental irradiations, including the exact phantom geometry and the RFCs, were simulated using the MCNP4C code. The origin of the polar coordinate system, (r,θ), was positioned at the center of the iridium core with $\theta =0^{\circ }$ corresponding to the side of the source's proximal end. The source was placed at the center of a $30\times 30\times 30\;{\text{cm}}^{3}$ cubic PMMA phantom. The ${\;}^{192}\text{Ir}$ photon spectrum used in this study was taken from the NuDat (National Nuclear Data Center) database.^(27)^ The procedures used for the extraction of the dose rate distributions were the same as the ones used by others.^16^, ^28^, ^29^ Accordingly, a grid system was used to estimate the dose rate distributions in PMMA which was composed of concentric annulus of 0.5 mm thickness with an angular distance of 1° (200×180=360,000 scoring cells), the absorbed dose was calculated in polar coordinates. To calculate the air‐kerma strength, the source was located in a $4\times 4\times 4\;m^{3}$ air volume with relative humidity of 40% (as recommended in the TG‐43U1). The air‐kerma strength, $S_{k}$, for IRAsource was scored along the transversal axis of the source using concentric annulus of 1 mm thickness, from r=99.5 cm to r=100.5 cm, or d=100 cm, where d is radial distance from the center of the radioactive source to the center of tally volume filled with air.

## III. RESULTS

**Figure 3:**
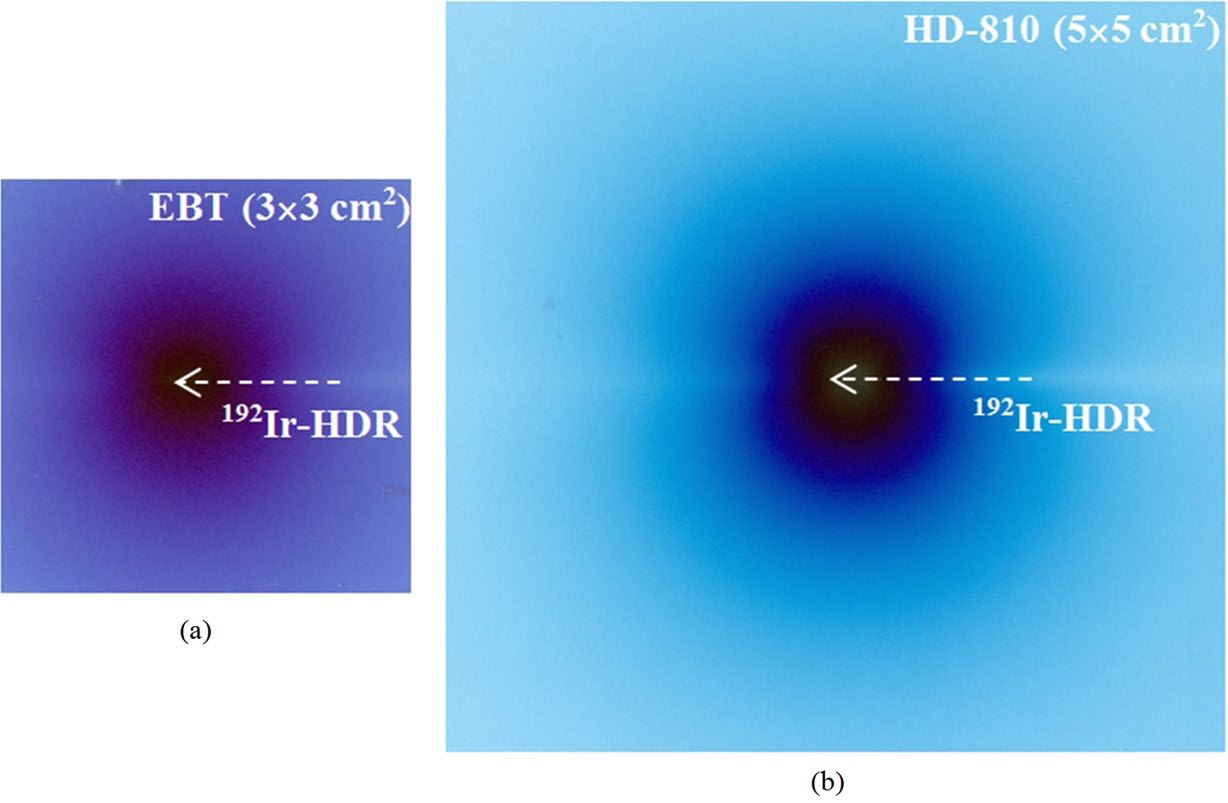
Scanned images of the (a) EBT $\left(3\times 3\;{\text{cm}}^{2}\right)$ and (b) HD‐810 $\left(5\times 5\;{\text{cm}}^{2}\right)$ RCFs, irradiated with an IRAsource‐HDR ${\;}^{192}\text{Ir}$, with activity of about 50 mCi, for 2 hr and 6 days, respectively.

**Figure 4:**
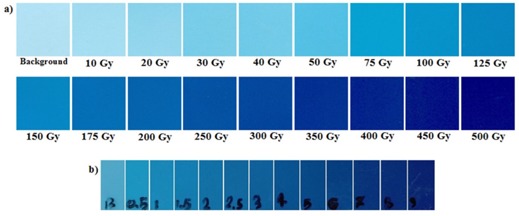
Scanned images of calibration and background films (a) HD‐810, (b) EBT.

Figure 3 shows the scan images of the EBT and HD‐810 RCFs exposed with IRAsource‐HDR ${\;}^{192}\text{Ir}$. It can be seen that the dose distribution of the HDR brachytherapy source has an elliptical shape. The scanned images of the calibrated and background films are shown in Fig. 4.

**Figure 5:**
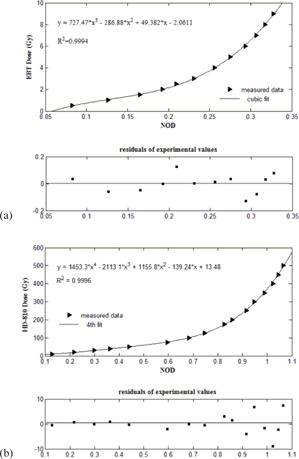
The dose response curves of (a) EBT and (b) HD‐810 RCFs, with fitting residue. The fitted function is shown as a solid line.

Figure 5 illustrates the calibration curves, net OD vs. dose (Gy), with fitting residue, plotted for HD‐810 and EBT RCFs. The calibration data were fitted with the third and fourth order polynomials for EBT and HD‐810 RCFs, respectively. The net ODs of experimental films were converted to dose in Gy using the fitted polynomials.

**Figure 6:**
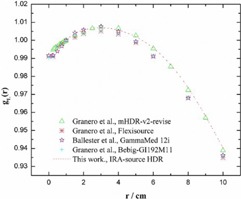
The radial dose functions of various ${\;}^{192}\text{Ir}$‐HDR sources.

The values of dose rate constant, Λ, in a unit of $\text{cGy}U^{-1}h^{-1}$ around the IRAsource‐HDR ${\;}^{192}\text{Ir}$ were found to be 1.084 and 1.129 using EBT and HD‐810 RCFs, respectively. Using MCNP4C code, the values of air‐kerma strength per unit activity, $S_{k}/A$, and the dose rate constant, Λ, were obtained to be $10.19\times 10^{-8}\;U\;{\text{Bq}}^{-1}$ and $1.112\;\text{cGy}\;h^{-1}U^{-1}$, respectively. All statistical uncertainties in MCNP4C simulations were below 0.5%. Table 2 compares the dose rate values obtained in this study with the corresponding values reported by others. The radial dose functions along the transverse axis of the IRAsource‐HDR ${\;}^{192}\text{Ir}$, at the radial distance of 0.5 to 4 cm, with the reference available data were tabulated in Table 3. Figure 6 compares the radial dose function obtained in this study (MC simulation) with other reported data at the radial distance of 0.5 to 10 cm.

**Figure 7:**
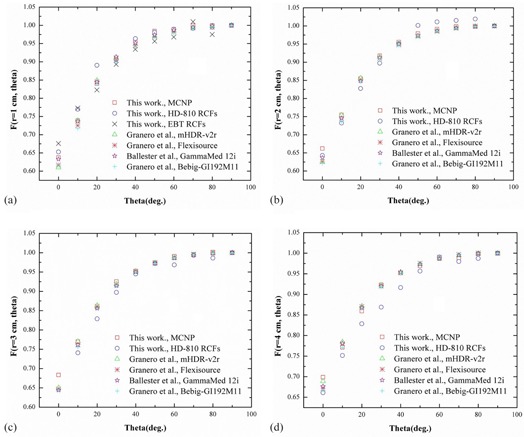
2D anisotropy function, F(r,θ), vs. the polar angle θ for various radial distances r, (a) r=1 cm, (b) r=2 cm, (c) r=3 cm, (d) r=4.

The 2D anisotropy functions, F(r,θ), were calculated at radial distances from 0.5 to 1.5 cm with EBT, and radial distances from 0.5 to 4 cm with HD‐810 and MC simulations in PMMA phantom. In Fig. 7 the calculated 2D anisotropy function was compared with the reference data.^13^, ^14^, ^15^, ^16^

**Table 2:** The dose rate constant values obtained in this study and those reported in the literature

*Source*	*Method*	Λ $\left({\text{cGyh}}^{-1}U^{-1}\right)$ Statistical Uncertainty (k=1)
mHDR‐v2r^(13)^	PENELOPE	1.112±0.0004
Flexisource^(14)^	GEANT4	1.109±0.011
GammaMed HDR 12i^(15)^	GEANT3	1.108±0.003
BEBIG GI192M11^(16)^	GEANT4	1.117±0.003
IRAsource (this work)	MCNP4C	1.112±0.008
IRAsource (this work)	HD‐810 film	1.129±0.044
IRAsource (this work)	EBT film	1.084±0.046

**Table 3:** The radial dose function calculated in this study, compared with other similar ${\;}^{192}\text{Ir}$‐HDR sources

*Radial Distance (cm)*	*HD‐810 RCFs (this work)*	*MCNP (this work)*	*Flexisource* ^(14)^	*mHDR‐v2r* ^(13)^	*GammaMed 12i* ^(15)^
1.0	1	1.0000	1.000	1.0000	1.000
1.5	1.0097	1.0027	1.002	1.0028	1.004
2.0	1.0147	1.0044	1.004	1.0050	1.006
2.5	1.0203	1.0054		1.0066	
3.0	1.0211	1.0050	1.005	1.0075	1.008
3.5	1.0195			1.0076	
4.0	1.0174	1.0019	1.003	1.0067	1.005

## IV. DISCUSSION

Uncertainties reported in TG‐43U1 are both random, statistical (type A) and nonrandom, systematic (type B). An estimate of the combined uncertainties has been calculated using a simple quadrature sum of individual components to provide an overall uncertainty. The detailed step‐by‐step analysis of the uncertainties of the measured doses is described by Chiu‐Tsao et al.^(1)^ According to MC simulations, the values obtained for type A uncertainty were less than 0.1% for all points except for the longitudinal axis (z‐axis) points where the statistical uncertainty was below 0.5%. The statistical uncertainty in the simulation of air‐kerma strength is less than 0.4%. This gives us a combined type A uncertainty of 0.41% for all points, except near longitudinal axis points where the combined type A uncertainty is about 0.64%. The type B uncertainty included uncertainties in cross‐sectional data, material definitions, source geometry and spectrum. The combined type B uncertainty is calculated to be about 0.71%. Thus, the overall A+B uncertainties for the MC simulations gives a combined uncertainty in the final dose rate values of 0.82% for all points except for the longitudinal axis points for which the overall A+B uncertainties are 0.96%.

The uncertainty analysis of the calibration is summarized in Table 4. The overall uncertainties in dose conversion were estimated, using a simple quadrature sum of individual components of Table 4, to be 4.1% and 3.9% for EBT and HD‐810 RCFs, respectively. The uncertainty analysis in the experimental procedure is summarized in Table 5. Likewise, the overall uncertainties of the dose rate constant were found to be 4.6% and 4.4% for EBT and HD‐810 RCFs, respectively.

According to the Table 2, the difference between the values obtained with MC simulation and RCFs measurements is equal to 2.5% and 1.5% for EBT and HD‐810 RCFs, respectively, and is within their uncertainties. Also, the maximum difference between the results of this study (MC) and other reference data is below 0.45%. It can be concluded with confidence that dose rate constant values obtained in this study are in good agreement with each other and also with published data.

For the radial dose function, the maximum difference between the MC results and HD‐810 measurements is 1.6% at the radial distance of 3 cm (Table 3). The maximum disagreement between the results of this research and other available data is observed to be 0.4% (Fig. 6).

From Fig. 7 it is evident that the results (2D anisotropy functions) are in good agreement with the reference data and MC results. The difference between measured data (EBT and HD‐810) and MC calculated is observed below 2%, for $\theta >20^{\circ }$. At $0<20^{\circ }$, in the zone close to the longitudinal axis of the source, the maximum difference observed is about 3.2%, due to the oblique filtration of the source.

In general, several factors including accelerator error in exposure, lack of uniform exposure to the film calibration, and the probability of film scratches/touching during the experiment cause errors in the RCFs measurement. Thus, the maximum disagreement between the MC data and the RCFs dosimetry is observed within 2%, for $\theta >20^{\circ }$. Also, the difference between the results of this study and the reference data is below 4%, which is due the physical differences in the construction of the source models.

**Table 4:** Uncertainty in dose conversion from optical density in the calibration procedure

	*EBT*		*HD‐810*	
*Uncertainty Description*	*Type A (%)*	*Type B (%)*	*Type A (%)*	*Type B (%)*
Film uniformity		2		3
Scanner consistency	1.5		1	
Fitted dose value process		2.5		1.5
Calibration film dose value (beam flatness)		2		1.8
Quadrature sum	1.5	3.77	1	3.8
Overall conversion uncertainty		4.1		3.9

**Table 5:** Uncertainty analysis for the experimental films

	*EBT*		*HD‐810*	
*Uncertainty Description*	*Type A (%)*	*Type B (%)*	*Type A (%)*	*Type B (%)*
Phantom full scatter (deviation from unbounded)	0.5		0.5	
Repeatability of film scanning	0.4		0.3	
Distance between source center and film emulsion		0.1		0.1
Source strength		2		2
Exposure time		0.05		0.02
Overall conversion uncertainty (from Table 4)		4.1		3.9
Quadrature sum	0.64	4.56	0.58	4.38
Overall uncertainty in dose rate		4.6		4.4

## V. CONCLUSIONS

Measured and calculated values of dose rate constant, radial dose function and 2D anisotropy function at radial distance of 0.5 to 4 cm for the first prototype IRAsource‐HDR${\;}^{192}\text{Ir}$ brachytherapy source in PMMA phantom are obtained in this study and compared with previously published data for interstitial ${\;}^{192}\text{Ir}$‐HDR sources. The statistical uncertainties of the MC, EBT, and HD‐810 dosimetry are calculated as 0.8%, 4.6%, and 4.4%, respectively. The maximum disagreement between the MC and the RCFs dosimetry results is observed within ±2%, at $\theta >20^{\circ }$ because of some uncertainties in RCFs measurements such as accelerator error in exposure, lack of uniform calibration film exposure, and the probability of film scratches/touching. At $\theta <20^{\circ }$, the difference is about ±3.2% because of the oblique filtration of the source. The results of this study agree within ±4.5% with the reference data available for interstitial ${\;}^{192}\text{Ir}$‐HDR sources. This deviation may partly be attributed to the physical difference in the construction of the source models.

The agreement between MC calculations and RCFs measurements suggests that HD‐810 RCF dosimetry can be used as an alternative to EBT RCF for HDR brachytherapy and justifies the use of specific data for this new source. These dosimetry parameters are essential, as they account for accurate determination of dose rate distribution around the brachytherapy source. Besides, they can be used as input data for the treatment planning systems used in HDR brachytherapy. By considering these parameters properly in the treatment planning systems, it is possible to improve the accuracy of the dose distribution curves.

## COPYRIGHT

This work is licensed under a Creative Commons Attribution 4.0 International License.
